# Delayed development of aphasia related to degeneration of the arcuate fasciculus in the dominant hemisphere nine years after the onset in a patient with intracerebral hemorrhage: a case report

**DOI:** 10.1186/s12883-021-02199-4

**Published:** 2021-04-20

**Authors:** Min Jye Cho, Sung Ho Jang

**Affiliations:** Department of Physical Medicine and Rehabilitation, College of Medicine, Yeungnam University, 317-1, Daemyungdong, Namku, Daegu, 705-717 Republic of Korea

**Keywords:** Diffusion tensor imaging, Neural degeneration, Aphasia, Arcuate fasciculus, Stroke

## Abstract

**Background:**

We report on a patient with an intracerebral hemorrhage (ICH), who showed delayed development of aphasia, which was demonstrated via follow up diffusion tensor tractography (DTT) to be related to neural degeneration of the arcuate fasciculus (AF).

**Case presentation:**

A 51-year-old, right-handed male presented with right hemiparesis, which occurred at the onset of a spontaneous ICH in the left corona radiata and basal ganglia. Brain magnetic resonance images showed a hematoma in the left subcortical area at one month after onset and hemosiderin deposits in the left subcortical area at nine years after onset. At four weeks after onset, he exhibited severe aphasia, and Western Aphasia Battery (WAB) testing revealed an aphasia quotient in the 39.6 percentile (%ile). However, his aphasia improved to nearly a normal state, and at three months after onset, his aphasia quotient was in the 90.5 %ile. At approximately eight years after onset, he began to show aphasia, and his aphasia increased slowly with time resulting in a WAB aphasia quotient in the 12.5 %ile at nine years after onset. The integrity of the left AF over the hematoma was preserved on 1-month post-onset DTT. However, the middle portion of the left AF in the middle of the hemosiderin deposits showed discontinuation on 9-year post-onset DTT. The fractional anisotropy value of the left AF was higher on the 9-year post-onset DTT (0.48) than that on the 1-month post-onset DTT (0.35), whereas the mean diffusivity value was lower on the 9-year post-onset DTT (0.10) than that on the 1-month post-onset DTT (0.32). The fiber number of the left AF was decreased to 175 on the 9-year post-onset DTT from 239 on the 1-month post-onset DTT.

**Conclusions:**

We report on a patient with ICH who showed delayed development of aphasia, which appeared to be related to degeneration of the AF in the dominant hemisphere. Our results suggest that DTT would be useful in ruling out neural degeneration of the AF.

## Background

Stroke is often accompanied by neural degeneration [1–3]. Several studies have reported stroke-related wallerian degeneration, which is characterized by degeneration of the nerve axon and its myelin sheath distal to the neural injury in the central and peripheral nervous system and starting immediately after neural injury in the acute stage of stroke [1–3]. However, little is known about delayed onset neural degeneration in stroke even though a diagnosis of delayed neural degeneration is clinically important because clinicians usually suspect recurrence of stroke when a stroke patient shows novel clinical manifestations after stroke onset [4].

Regarding intracerebral hemorrhage (ICH), the possibility of neural degeneration due to chemical injury by hematomal material has been suggested [5–9]. Since the introduction of diffusion tensor imaging (DTI), a few studies have applied DTI in patients with ICH to detect delayed onset neural degeneration in neural tracts such as the corticospinal, corticoreticulospinal, and spinothalamic tracts [7–9]. However, no study on neural degeneration of the arcuate fasciculus (AF), a neural pathway that connects Wernicke’s area to Broca’s area, has been reported [10].

In the current study, we report on a patient with ICH who showed delayed development of aphasia due to neural degeneration of the arcuate fasciculus (AF), which was demonstrated by performing follow up diffusion tensor tractography (DTT).

## Case presentation

**Fig. 1:**
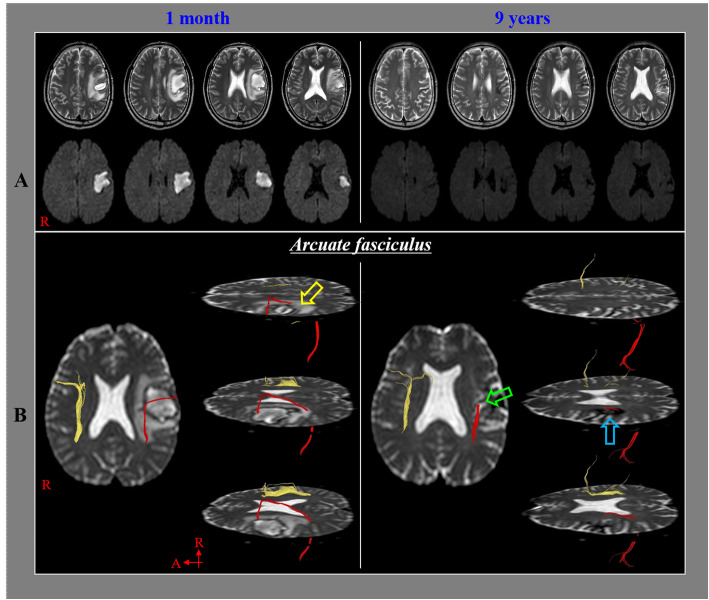
**a**. T2-weighted and diffusion-weighted brain magnetic resonance images show hematoma presence in the left subcortical area at one month after stroke onset and hemosiderin deposits in the left subcortical area at nine years after onset. **b**. The integrity of the left arcuate fasciculus was preserved through the hematoma (yellow arrow) on 1-month post-onset diffusion tensor tractography. However, the middle portion of the left arcuate fasciculus showed discontinuation (green arrow) in the middle of an area of hemosiderin deposits (sky-blue arrow) on 9-year post-onset diffusion tensor tractography.

A 51-year-old, right-handed male presented with right hemiparesis, which presented at the onset of a spontaneous ICH in the left corona radiata and basal ganglia [11]. Brain magnetic resonance images were obtained four weeks after onset of the ICH showed a leukomalactic lesion in the left corona radiata (Fig. 1-a). At four weeks after onset, he showed severe aphasia and Western Aphasia Battery (WAB) testing revealed an aphasia quotient in the 39.6 percentile (%ile; fluency: 17.0 %ile, comprehension: 81.0 %ile, repetition: 21.0 %ile, and naming: 57.0 %ile) [12]. His aphasia improved to nearly a normal state at three months after ICH onset when his aphasia quotient was in the 90.5 %ile (fluency: 88.6 %ile, comprehension: 88.2 %ile, repetition: 66.3 %ile, and naming: 98.4 %ile). At approximately eight years after onset, he began to show aphasia and his aphasia worsened slowly with time without the occurrence of a new neurological disease. At nine years after ICH onset his WAB results indicated an aphasia quotient in the 12.5 %ile (fluency: 9.0 %ile, comprehension: 91.1 %ile, repetition: 9.0 %ile, and naming: 9.0 %ile). The patient provided written informed consent prior to starting this study, and the study protocol was approved by the institutional review board of a university hospital.

### Diffusion tensor imaging

The DTI was performed twice (at one month and nine years after ICH onset). On both occasions, a sensitivity-encoding head coil on a 1.5-T Philips Gyroscan Intera scanner (Hoffman-LaRoche Ltd., Best, Netherlands) with single-shot echo-planar imaging and navigator echo was used for the acquisition of DTI data.67 contiguous slices were obtained parallel to the anterior commissure–posterior commissure line for each of the 32 non-collinear diffusion-sensitizing gradients [13]. DTI parameters were as follows: acquisition matrix = 96 × 96; reconstructed matrix = 128 × 128; field of view = 221 mm × 221 mm; repetition time = 10,726 ms; echo time = 76 ms; parallel imaging reduction factor = 2; echo-planar imaging factor = 49; b = 1000 s/mm^2^; number of excitations = 1; and slice thickness of 2.3 mm with no gap (acquired voxel size 1.25 mm × 1.25 mm × 2.5 mm) [13]. By using the fiber assignment continuous tracking algorithm, fiber tracking was performed within the diffusion tensor imaging task card software (Philips Extended MR Work Space 2.6.3, Philips, Amsterdam, Netherlands), and the DTI parameters (fractional anisotropy [FA], mean diffusivity [MD], and fiber number [FN, total voxel number of a neural tract]) were automatically calculated using PRIDE software (Philips Medical Systems, Best, Netherlands) [13]. Image distortions and head motion effects was corrected by using a diffusion registration package (Philips Medical Systems) [13]. For analysis of the AF, the seed ROI was assigned manually in the deep white matter located in the posterior parietal portion of the superior longitudinal fascicle, while the target ROI was placed on the posterior temporal lobe using the method of Nucifora et al. [14]. The fiber tracts were visualized due to the target of interest passing through the two ROIs. The termination criteria used for fiber tracking were a FA value < 0.15 and an angle < 27^o^.

The integrity of the left AF over the hematoma was observed to be preserved on the 1-month post-onset DTT (Fig. 1-b). However, the middle portion of the left AF in the middle of the area with hemosiderin deposits showed discontinuation on the 9-year post-onset DTT. The FA value of the left AF was higher on the 9-year post-onset DTT (0.48) than that on the 1-month post-onset DTT (0.35), whereas MD value was lower on the 9-year post-onset DTT (0.10) than that on the 1-month post-onset DTT (0.32). On the other hand, the FA value of the right AF was higher on the 1-month post-onset DTT (0.441) than that on the 9-year post-onset DTT (0.405), whereas the MD value was lower on the 1-month post-onset DTT (0.219) than that on the 9-year post-onset DTT (0.381). The FNs of the left AF and the right AF were decreased to 175 and 617 on the 9-year post-onset DTT from 239 to 1149 on the 1-month post-onset DTT, respectively.

## Discussion and conclusions

Based on the results obtained, we suggest that the delayed development of this patient’s severe aphasia was related to delayed neural degeneration of the AF in the dominant hemisphere. Our rationale is based on the following observations. The patient showed aphasia (aphasia quotient: 39.6 %ile) at ICH onset; however, his aphasia had recovered to a nearly normal range (aphasia quotient: 90.5 %ile) at three months after onset. The integrity of the left AF just above the hematoma in the dominant hemisphere was shown to be preserved on the 1-month post-onset DTT (Fig. 1-b). However, he began to develop aphasia approximately eight years after onset, and his aphasia worsened slowly to a level at which he could not communicate via speech at nine years after onset (aphasia quotient: 12.5 %ile). On DTT at 9-year post-onset, there was a discontinuation of the middle of the left AF, and hemosiderin deposits were observed around the discontinued portion (Fig. 1-b). The FA of the left AF was higher on the 9-year post-onset DTT than that on the 1-month post-onset DTT. By contrast, the values of MD and FN were lower on the 9-year post-onset DTT than that on the 1-month post-onset DTT. The FA value represents the degree of directionality of water diffusion and integrity of white matter microstructures, such as axons, myelin, and microtubules [15, 16]. The MD value suggests the magnitude of water diffusion, whereas the FN indicates the total number of neural fibers in a neural tract [15, 16]. As a result, the increased FA and decreased MD values of the left AF represent increased directionality and decreased magnitude of water diffusion respectively, and the decreased FN indicates decreased voxel numbers of the left AF [15–17]. Our results with regard to change of FA value could suggest that the left AF was affected by selective degeneration induced by lower neural branching, reduced axon diameter, or increased coherence of intact neural fibers connection. The results appeared to be coincided with the previous studies which have reported that neural degeneration resulted in increased FA value in patients with multiple sclerosis, Williams syndrome, and Parkinson’s disease [18–20]. On that basis, we think that the delayed development of aphasia of this patient was related to a delayed degeneration of the left AF; however, when this degeneration began is unclear. Regarding the pathophysiological mechanism of neural degeneration of the left AF, previous studies have suggested a chemical causative mechanism. That mechanism is based on reports that a blood clot can cause extensive damage to neural tissue by releasing potentially damaging substances, such as free iron, which may generate the release of free radicals or inflammatory cytokines [5–9]. We suggest that although the left AF was shown to have successfully passed through the hematoma on the 1-month DTT, there was a discontinued portion of the left AF in the middle of an area of hemosiderin deposits on the 9-year DTT. This discontinuation appears to be related to the above chemical-based pathophysiological mechanism of delayed neural degeneration in this patient. On the other hand, the result that the FN of the right AF was also decreased during nine years might be caused by aging or disuse atrophy due to severe aphasia. Consequently, we think there was a possibility that the left AF might be degenerated by aging.

In conclusion, we report on a patient with ICH who showed delayed development of aphasia that may be related to degeneration of the AF in the dominant hemisphere. Our results suggest that DTT can be useful in ruling out neural degeneration of the AF. To the best of our knowledge, this is the first study to report on delayed neural degeneration of the AF nine years after the onset in a patient with ICH [7–9]. However, because it is a case report, this study has limitation; therefore, the conduct of additional complementary studies involving larger case numbers is warranted. The another limitation is lack of serial clinical and radiological data during nine years. In addition, limitation of DTT should be considered: results of DTT can be false positive or negative due to crossing fiber and partial volume effects [21].

## Data Availability

The datasets generated and/or analyzed during the current study are not publicly available due to institutional restrictions but are available from the corresponding author on reasonable request.

## Abbreviations

ICH: Intracerebral hemorrhage
DTI: Diffusion tensor imaging
AF: Arcuate fasciculus
DTT: Diffusion tensor tractography
WAB: Western Aphasia Battery
FA: Fractional anisotropy
MD: Mean diffusivity
FN: Fiber number
